# Investigating reproductive success of the ladybird beetle *Harmonia axyridis* from the perspective of micropyle variation

**DOI:** 10.1038/s41598-019-49249-z

**Published:** 2019-09-04

**Authors:** Yuan-Xing Sun, Ya-Nan Hao, Chang-Zhong Liu, Sen-Shan Wang

**Affiliations:** College of Plant Protection, Gansu Agricultural University/Biocontrol Engineering Laboratory of Crop Diseases and Pests of Gansu Province, Lanzhou, Gansu 730070 China

**Keywords:** Behavioural ecology, Entomology

## Abstract

Micropyles in insects are small openings that allow sperm entry into, and the number was usually decreased on unfertilized and (or) undeveloped eggs. However, reports showed that *Harmonia axyridis*, a reproductive success model, deposited similar number of micropyles on undeveloped and developing eggs. Thus, it was confusing whether micropyles in *H. axyridis* were unaffected. To solve this confusion, two experiments were conducted here. Firstly, virgin female and four different days delayed mating (DDM) experiments were conducted to reveal the effects of fertilization stimulus and delayed-fertilization. Secondly, intercrosses between a light-colored mutant (HAM, an adaptive deficiency) and wild type (HAW) were conducted to further reveal whether there were female and male interactions. We found that (1) eggs produced by virgin and DDM females had significantly less micropyles than control. Even so, more than 18 micropyles were observed on eggs following fertilization and, consequently, egg production as well as hatch rate was not negatively affected by mating delay; (2) number of micropyles was significantly varied among the four reciprocal crosses and virgin HAW female. Specifically, the heterozygous eggs (Cross-D) and wild-type homozygous eggs (Cross-B) respectively had the least and maximum micropyles, and eggs from virgin HAW female had significantly less micropyles compared to those from HAW female (Cross-B or Cross-C), but the number was significantly higher than those from HAM female (Cross-A or Cross-D). These results informed us that the number of micropyles in *H. axyridis* is plastic but maintaining a high-quantity that offers many benefits, which should contribute to its reproduction success.

## Introduction

In insects, there were one or more small openings locating on the eggshell that called micropyles^1^. Each micropyle was a channel that extended from the external gateway through chorion and ended in vitelline membrane, serving as the route of sperm entry into a mature oocyte^2,3^. In addition, for some species, there were special accessory structures located in micropyle region facilitating sperm penetration^4^. Thus, the number of micropyles as well as their structures had crucial roles in the fertilization of eggs^5^. At the functional level, eggs equipped with multiple micropyles would suggest greater potential for multiple sperm entry into an egg, which might offer several benefits, e.g. increase the opportunity for post-copulatory choice of female within the egg environment^6^.

However, the formation of micropyles was a considerable material and (or) energetic cost process^7^. Thus, for some insect species, the number of micropyles could be modulated on females’ own accord, which acted as a critical adaptive mechanism. For some species laying both viable and inviable eggs, the number of micropyles was vastly different between these two types^8^. Among which, the subsocial burrower bug, *Adomerus triguttulus* (Heteroptera: Cydnidae) and the stingless bee, *Trigona* (*Tetragonisca*) *angustula* (Hymenoptera: Apidae), were two typical examples. Their viable eggs were presented with micropyles, whereas their inviable eggs (trophic eggs) were by and large devoid of such structures^7,9^. The above cases might be caused by the fact that inviable eggs were usually produced as food for newly hatched larvae, which could ensure the offspring have siblings to eat^10^. In addition, the number of micropyles also showed to be influenced by the experience of copulation (or called fertilization). For example, virgin female workers of the ant, *Gnamptogenys menadensis* (Hymenoptera: Formicidae) and virgin queen of the subterranean termite, *Reticulitermes chinensis* (Isoptera: Rhinotermitidae) can only lay small number of eggs lacking micropyle; while those females following mating produced a mass number of eggs presenting micropyles^11,12^. Queens of the termite, *Reticulitermes speratus* (Isoptera; Rhinotermitidae), in particular, could close the gate of micropyle to switch from sexual to asexual reproduction when the kings were presented^13^. The results from above studies indicated that the number of micropyles in some insect species is plastic. However, most studies only focused on the viable and inviable eggs that might be too limited to elucidate the variation as well as function of micropyles.

The multicolored Asian ladybird beetle, *Harmonia axyridis* (Coleoptera: Coccinellidae), native to Asia, is an important biological control agent and has been introduced to many areas. However, this ladybird beetle quickly became invasive and spread rapidly worldwide due to its excellent reproductive success^14,15^. Previous studies showed that female *H. axyridis* could also lay developing and undeveloped eggs (trophic eggs) synchronously, and mother ladybird beetle could adjust the proportion of undeveloped trophic eggs to mitigate the starvation risk of offspring^16^. However, similar number of micropyles (about 19.8) was detected on the developing and undeveloped eggs^5^. Thus, it was confusing whether the number of micropyles in *H. axyridis* was unaffected, and, if the number was plastic, what degree could be affected. In this study, we desinged two experiments to evaluate the variation of micropyles in *H. axyridis*. (1) Delayed mating is common in nature due to many factors, e.g. temperature, rain or wind^17^, and studies showed that delayed mating usually caused detrimental effects on female reproduction^18–20^. For *H. axyridis*, the first copulation has been confirmed to be the stimulus for normal oviposition. The virgin females could only lay a few scattered eggs, whereas they laid many eggs in batches soon after the first copulation^21^. Here, we hypothesized that the number of micropyles as well as the reproductive output in *H. axyridis* could be affected by the delay of first copulation/fertilization. (2) In insects, the mutations usually had vastly different biological and physiological characteristics compared to the wild type. Studies on *Bombyx mori* (Lepidoptera: Bombycidae) have shown that the eggs of mutations (e.g. *emi, Ge, Se*, *bd* and *bd*^sw^) usually had vastly different number of micropylar channels compared to those of normal strain^22–24^. For *H. axyridis*, a light-colored mutant *gr* (HAM) was found in our laboratory and displayed obvious different elytra coloration (grey-dark spots) compared to the wild type (HAW, deep-dark spots), and also showed to be an adaptive deficiency^25^. Thus, we hypothesized that the number of micropyles in HAM might be different from that in HAW. In addition, phenotype-dependent mate choice has been confirmed to be obvious in *H. axyridis*. For example, in a Chinese population, both *succinic* and *melanic* females preferred to mate with *succinic* males and resulted in relatively higher fertility, which showed that melanism in *H. axyridis* might result in pleiotropic effects on male fertility^26^. Here, we doubt if there were any significant differences of the number of micropyles among the crosses of HAM and HAW. Actually, studies conducted before have shown that mysterious interaction effects of HAM and HAW were detected for egg production as well as hatch rate^25^.

In this study, a series of delayed mating experiments were conducted to confirm whether fertilization stimulus as well as mating delay would affect the number of micropyles as well as reproductive output. After that, a reciprocal cross experiments of HAM and HAW were conducted to confirm whether there was any significance of the number of micropyles between HAM and HAW, in particular, focused on the effects of male partner. The aim of this study was to elucidate the reproductive characters of *H. axyridis* from the perspective of micropyle variation, especially under delayed mating conditions.

## Results

### Delayed mating experiment

#### Number of micropyles

**Figure 1:**
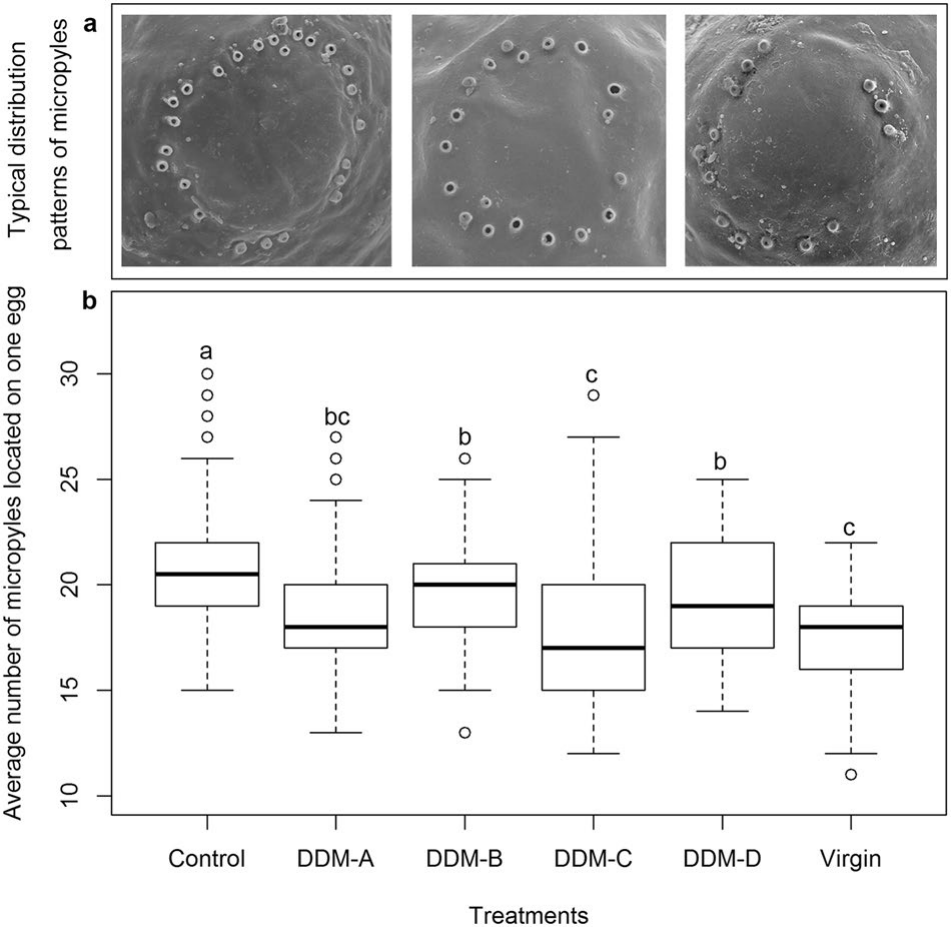
Typical distribution patterns of micropyles (**a**), and the average number of micropyles located on an egg produced by virgin female and delayed mating females (**b**). In the box plots, horizontal line within the box is the median; box indicates the lower and upper quartiles; capped vertical lines are 95% confidence limits, and white dots are outliers. Different letters indicate significant differences (Kruskal-Wallis test, *p* < 0.05). DDM-A, DDM-B, DDM-C, and DDM-D respectively represented treatment 2, 7, 12, and 17 days of mating delay.

Typical distribution patterns of the micropyles located on an egg were shown in Fig. 1a. Eggs produced by females in control had an average number of 21.0 ± 0.4 micropyles, which was significantly higher than that in treatment virgin female (17.6 ± 0.3) and those in the four delayed mating treatments (DDM-A (18.6 ± 0.3), DDM-B (19.6 ± 0.3), DDM-C (18.0 ± 0.4) or DDM-D (19.6 ± 0.3)) (Kruskal-Wallis χ^2^ = 67.358, *p* < 0.0001). Number of micropyles deposited on eggshell in treatments DDM-B and DDM-D was significantly higher than that in treatment virgin female; while, no significant difference was detected among the four delayed mating treatments, except that DDM-C had significantly less micropyles compared to DDM-B or DDM-D (Kruskal-Wallis χ^2^ = 67.358, *p* < 0.001) (Fig. 1b).

#### Egg production

**Figure 2:**
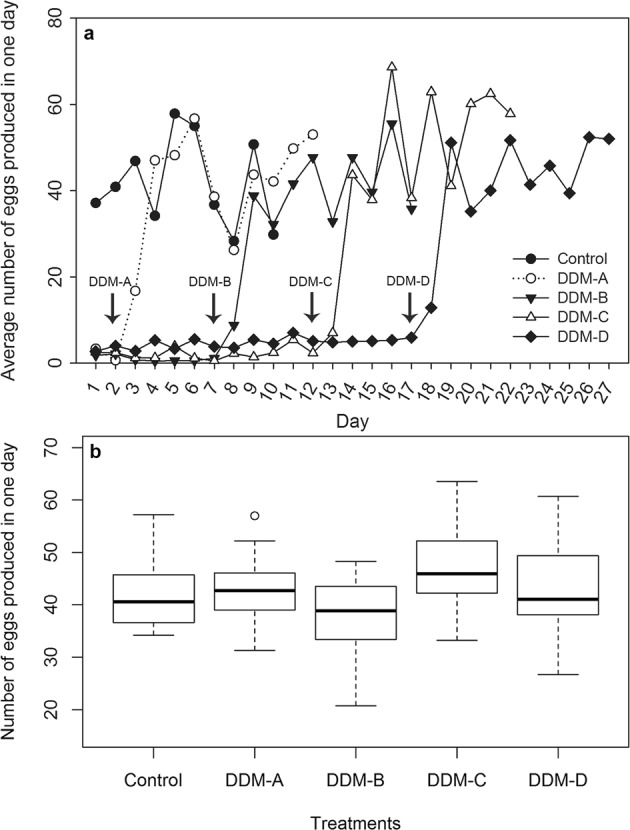
Dynamic trend of the egg production of virgin female and four delayed mating treatments (**a**), and the average number of eggs produced by the delayed mating and normally mated (control) females (**b**). a: the arrow indicated the date that provided a male for virgin female in treatments DDM-A, DDM-B, DDM-C, and DDM-D. b: in the box plots, horizontal line within the box is the median; box indicates the lower and upper quartiles; capped vertical lines are 95% confidence limits, and white dots are outliers. No significant differences were detected among the treatments (Tukey HSD test, *p* > 0.05). DDM-A, DDM-B, DDM-C, and DDM-D respectively represented treatment 2, 7, 12, and 17 days of mating delay.

Virgin female of *H. axyridis* can only lay small number of eggs on each day; while the oviposition greatly increased at the third day following virgin female mated with a male, irrespective of the female age at mating (Fig. 2a). For the 10 days’ post-mating oviposition, the average number of eggs produced by female was similar in treatment DDM-A (43.2 ± 2.3), DDM-B (37.7 ± 2.6), DDM-C (47.4 ± 2.9), and DDM-D (43.3 ± 3.1), and all were not significantly different from that in control (42.3 ± 2.3) (*F*_4, 45_ = 1.672, *p* = 0.173) (Fig. 2b).

#### Egg hatch rate

**Figure 3:**
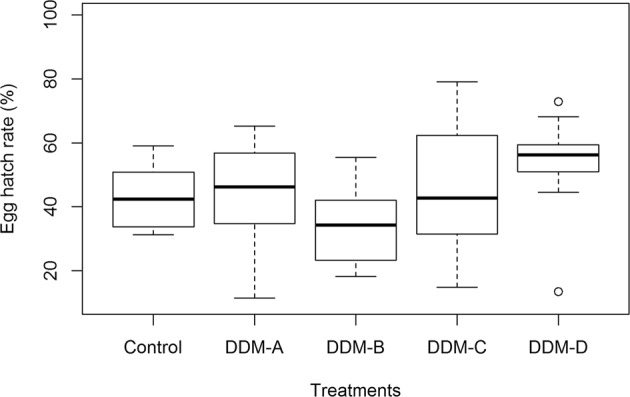
Box plots showing differences in egg hatch rates among the four delayed mating treatments and control. In the box plots, horizontal line within the box is the median; box indicates the lower and upper quartiles; capped vertical lines are 95% confidence limits, and white dots are outliers. No significant differences were detected among the treatments (Kruskal-Wallis test, *p* > 0.05). DDM-A, DDM-B, DDM-C, and DDM-D respectively represented treatment 2, 7, 12, and 17 days of mating delay.

Egg hatch rates were also not significantly affected by delayed mating. No significant differences were detected among the four delayed mating treatments (DDM-A (45.2 ± 5.2%), DDM-B (34.1 ± 3.8%), DDM-C (45.4 ± 6.8%), and DDM-D (53.2 ± 5.1%)), and all were similar to that in control (43.0 ± 3.1%) (Kruskal-Wallis χ^2^ = 8. 889, *p* = 0.064) (Fig. 3).

### Crossing experiments of the mutant and wild phenotype

**Figure 4:**
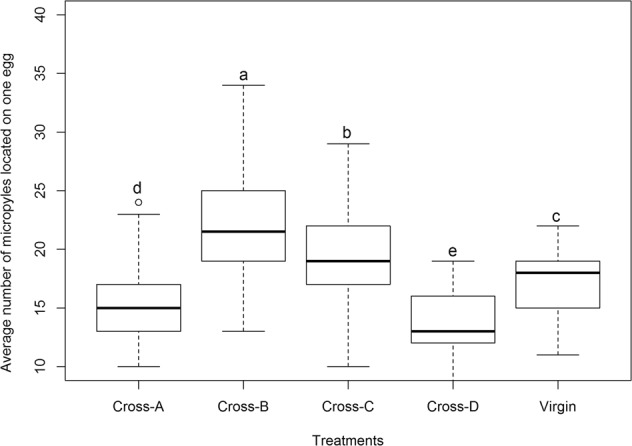
Box plots showing differences in average number of micropyles among the four crosses of light-colored mutant of *H. axyridis* (HAM) and wild type *H. axyridis* (HAW). In the box plots, horizontal line within the box is the median; box indicates the lower and upper quartiles; capped vertical lines are 95% confidence limits, and white dots are outliers. Asterisk indicates a significant difference (Kruskal-Wallis test, *p* < 0.05). Cross-A, Cross-B, Cross-C, and Cross-D respectively represented the cross ♀(HAM) × ♂(HAM), ♀(HAW) × ♂(HAW), ♀(HAW) × ♂(HAM), and ♀(HAM) × ♂(HAW).

The average number of micropyles located on an egg varied significantly among the four crosses, and they all were significantly different from that of virgin HAW female (Kruskal-Wallis χ^2^ = 187.09, *p* < 0.001). Specifically, among the four crosses, number of micropyles located on wild-type homozygous eggs from Cross-B (22.1 ± 0.5) was significantly higher than those on eggs from the other three crosses, and the least number was detected on heterozygous eggs from Cross-D (13.6 ± 0.3); the mutant homozygous eggs from Cross-A (15.2 ± 0.3) had significantly higher number of micropyles compared to the heterozygous eggs from Cross-D, but the number was significantly smaller than those on heterozygous eggs from Cross-C (19.1 ± 0.4). Eggs from virgin HAW female had significantly smaller number of micropyles (17.4 ± 0.3) compared to those from HAW female in Cross-B or Cross-C, but the number was significantly higher than those from HAM female in Cross-A or Cross-D (Kruskal-Wallis χ^2^ = 187.09, *p* < 0.001) (Fig. 4).

## Discussion

In this study, we found that the number of micropyles in *H. axyridis* was greatly affected by the fertilization stimulus of female, and delayed mating (or fertilization) can also result in the variation of micropyles. Based on the mutation HAM, we further found that female as well as its interaction with male partner can determine the number of micropyles. All these results proved that the number of micropyles in *H. axyridis* was plastic.

In delayed mating experiments, virgin female of *H. axyridis* can lay small number of eggs that were also presented with micropyles, but the average number was significantly smaller than that in control. These results indicated that the stimulus of copulation (or special substances) might affect the formation of micropyle on eggshell. The possible mechanisms might be listed as following. Firstly and most importantly, the formation of micropyles was a considerable material and (or) energetic cost process^7^, while for virgin female, the micropyles serving as the route of sperm entry into a mature oocyte was not functional necessary^2,3^. Thus, decreasing the number of micropyles on eggs produced by virgin female might be a material and (or) energetic saving strategy. In addition, in the process of internal fertilization, females obtained a cocktail of molecules which could affect virtually all aspects of the female’s reproductive activity, e.g. leading to the increasing production of yolk protein by follicle cells^27–30^, and, during insect oogenesis, micropyles has been confirmed to be deposited by the follicle cells^31^. Thus, virgin female that not obtain such molecules from male might affect the formation of micropyles. The typical examples were observed in many social insect species (e.g. ant or termite) that the micropyle structure disappeared on eggs produced by virgin workers^11,12^. Here, for *H. axyridis*, almost 17 micropyles were still presented on eggs from virgin female. Results from delayed mating experiments partly revealed the significance of this specialty and the crucial roles of timely fertilization in normal formation of micropyles.

When the virgin female was provided with a male at different periods post the first oviposition, the average number of micropyles located on an egg was still significantly less than that in control. For *H. axyridis*, Obata^21^ deduced that their mating acceptance is coincides with the start of oocyte development, and studies showed that the formation of egg shell and other associated structures is occurred at around the 6–7^th^ day post-emergence under 25 °C^32^. Under similar developmental temperature, first mating of *H. axyridis* occurred at the 3^rd^ day post-emergence and all individuals have mated at least one time at the 5^th^ day^33^. Thus, mating occurring of *H. axyridis* seemed to be a little earlier than eggshell formation, and timely copulation/fertilization might be the stimulus for normal formation of micropyles on eggshell. However, in a social insect, the honey bee, *Apis mellifera* (Hymenoptera: Apidae), the change in micropylar chorionic morphology was reported to be fertilization-independent^34^. In this study, we also found that eggs produced by females with specific periods of delayed mating (e.g. DDM-B and DDM-D) had significantly more micropyles than that in virgin female. Osawa^35^ reported that the ovarian development of *H. axyridis* was selection pressure-dependent and showed to be dynamic in ways that permit populations to cope with exotic variations (e.g. host and food resources). In our delayed mating experiments, the great increasing of egg production occurred two days later after the virgin female was mated. Thus, the differences of micropyles between DDM-B (or DDM-D) and virgin female might be caused by the come across of copulation/fertilization and special ovarian development stage of virgin female that following 7th or 17th day of asexual oviposition, but the real mechanisms remain still unclear.

In the intercross experiments of HAM and HAW, we found that the phenotype (or called quality) of female and male can also greatly affect the number of micropyles. Results showed that significantly less micropyles was detected on the mutant eggs from Cross-A compared to homozygous wild-type eggs from Cross-B. More importantly, mutant homozygous eggs from Cross-A or heterozygous eggs from Cross-D that produced by female HAM even had significantly smaller number of micropyles than the unfertilized eggs from virgin HAW female. These results indicated that the function of special genetic process might be dominated over that of fertilization. In insects, exochorion formation is governed by the genetic processes occurring within the follicle cells^36^. For example, the gene hemipterous (*hep*) is required for morphogenesis of micropyles in *Drosophila*^37^; and in the German cockroach, *Blattella germanica* (Blattaria: Blattellidae), the gene *Brownie* is necessary for the formation of micropyle-like structures^38^. Here, HAM is a single gene mutation that related to body pigmentation and how such pleiotropic effects occurring remain unknown. More importantly, the significant variances detected among the four crosses indicated a mysterious female × male interaction between HAM and HAW. For many insect species, female usually exhibit different reproductive investments in response to male quality^39^. HAM has been confirmed to be an adaptive deficiency^25^, and thus selective investments in reproduction might be occurred in the intercross of HAM and HAW. Actually, similar phenomenon was also found in egg production and hatch rate^25^.

However, in any case, a large quantity of micropyles were detected on eggs that produced by female *H. axyridis*. At a functional level, more micropyles would suggest greater potential for multiple sperm entry into an egg, which might offer several benefits. Firstly, the presence of multiple micropyles could increase the opportunity for post-copulatory choice of female within the egg environment^6^. For example, in the polyspermic ctenophore, *Beroe ovata*, the female pronucleus migrates among male pronuclei within the egg before fusing with one^40^, and studies conducted in Lepidoptera have also confirmed that the micropyle number was positively correlated with female promiscuity^6^. Actually, for *H. axyridis*, multiple mating is prevalent^41^, and the sperm stored by a female was from multiple males^42^. Secondly, polyspermy might be important for embryonic development. Studies conducted in both domestic fowl and the zebra finch, *Taeniopygia guttata* have confirmed that polyspermy is essential for their early embryonic development^43^. However, whether such deduced benefits are all available for an insect *H. axyridis* requires further studies to confirm, and other possible benefits also need to be explored.

In this study, mating delay, at least, did not cause any negative effects on the reproductive performance (egg production and hatch rate) of female *H. axyridis*; while, on the contrary, almost all studies conducted before have reported that delayed mating would result in reduced fecundity and fertility^20,44,45^. Maintaining high reproductive capability under mating delay conditions might be important characters contribute to the reproduction success of *H. axyridis*. Firstly, it can ensure this ladybird beetle overcome the negative effects caused by wind, rain or unfavorable temperatures during mating stage^17^. In addition, studies showed that *H. axyridis* females mostly overwintered (up to several months) as unmated individuals^46,47^, and the physiological status of non-mate could also greatly increase the survival of post-storage adults^48^. The characteristic that maintaining high reproductive output under mating delay could help them to quickly establish populations after winter season. In practice, based on these specialties, non-mated individuals of *H. axyridis* would be suitable for cold storage, and thus could further promote their application in biological control program in the native regions.

In conclusion, our results confirmed that the number of micropyles in *H. axyridis* is plastic, but maintaining a high-quantity that would offer many benefits. In perspective of micropyle, the results revealed another possible mechanism that contributes to the reproduction success of *H. axyridis*. In addition, these results informed us new knowledge of factors that could influence the formation of micropyle in insects, which might encourage more studies to elucidate the molecular mechanisms.

## Materials and Methods

### Insects

Colonies of the pea aphid (*Acyrthosiphon pisum* (Hemiptera: Aphididae)) and the peach aphid (*Myzus persicae* (Hemiptera: Aphididae)) were respectively established on broad bean (*Vicia faba*, var. “Jinnong”) and pepper (*Capsicum annum* fasciculatum, var. “Changfeng”) seedlings in nylon mesh cages (60 × 45 × 40 cm). The wild type of *H. axyridis* (indicated as HAW) were from continuous laboratory rearing colonies established on *A. pisum*; while, the colony of light-colored mutant of *H. axyridis* (HAM) was established on *M. persicae* due to their poor reproductive performance on *A. pisum* (unpublished data). All these insects were reared in an insectary (24 ± 1 °C, 65% RH and 14:10 h L: D) at Gansu Agricultural University.

In order to prepare newly emerged adults for subsequent experiments, two pairs of HAW and HAM were respectively kept in a plastic Petri dish (9 cm in diameter) and supplemented with sufficient aphids as food. In addition, two host plant leaves were provided as the oviposition substrate. The egg production was checked once a day and the eggs were incubated in a new plastic Petri dish (3 cm in diameter) with an immersed cotton ball for keeping moisture. Newly hatched larvae (25 to 30) were reared in plastic Petri dishes (9 cm in diameter, 1.5 cm in height) equipping with barriers, which was confirmed to be effective in reducing larval cannibalism (unpublished data). Specifically, the barriers were made with transparent plastic bands (1.5 cm in width), and one circular band (4.5 cm in diameter) was connected with eight short bands (4.5 cm long) with equal distance (one end of them were jointed together in the center area of the Petri dish). Thus, the Petri dish area was divided into 16 partitions, while the larvae could move freely through a small gap located in the walls of each partition. The larvae were daily supplied with sufficient *A. pisum* (for HAW) or *M. persicae* (for HAM) (ca 120 mg) as food until pupation. Adult emergence was monitored and the newly emerged adults were used for different measurements. The following experiments were all conducted in bioclimatic chambers set at 24 ± 1 °C, 65% RH and 14:10 h L:D.

### Experimental design and sample collection

#### Delayed mating experiments

Newly emerged HAW adults were sexed and equally divided into six groups. Group 1, female was immediately paired with a male after emergence and served as control. Group 2, continuous egg production by virgin females, in which the female adults were individually reared throughout the experiments. The rest adults were individually reared in plastic Petri dishes (9 cm in diameter) for treatments of mating delay. Egg production of all females was carefully checked once a day. We found that females in control as well as the virgin females started to lay eggs at almost the 7^th^ day post-emergence. Once egg production initiated, the virgin females were divided into four groups. Group 3–6, different days delayed mating (DDM), each female was provided with a male (no mating experience) at the 2^nd^, 7^th^, 12^th^, or 17^th^ day post the first oviposition and respectively named DDM-A, DDM-B, DDM-C, and DDM-D. Eggs from each pair were counted and incubated in one Petri dish (3 cm in diameter) with an immersed cotton ball for keeping moisture. Specially, at the 2^nd^, 5^th^, and 10^th^ day post-mating, 6–8 eggs were randomly selected from the egg cluster of each pair and used for determination of the number of micropyles. After hatching, the inactive infant larvae were counted and immediately transferred out from the Petri dish to avoid cannibalizing on the eggs. Egg hatch rate was calculated following the equation: egg hatch rate = number of hatched larvae/number of eggs incubated × 100%. In total, 10 females were used for each treatment, and their post-mating oviposition were recorded for 10 days.

#### Crossing experiments of HAM and HAW

Newly emerged adults of HAM and HAW were paired as four groups with the design of reciprocal crosses, and each pair were kept in a plastic Petri dish and supplied with sufficient *M. persicae* (ca 100 mg) infesting on pepper leaves. The crosses ♀(HAM) × ♂(HAM), ♀(HAW) × ♂(HAW), ♀(HAW) × ♂(HAM), and ♀(HAM) × ♂(HAW) were respectively named as Cross-A, Cross-B, Cross-C, and Cross-D. Their oviposition were checked once a day and, at the 5–6^th^ day post-oviposition, 15 eggs were randomly selected from the egg cluster of each pair for determination of the number of micropyles. In total, twelve pairs were used for each group.

#### Determination of the number of micropyles

After collection, the eggs from each pair were immediately transferred to a 1.5 ml centrifuge tube containing 10% ethanol. Then, they were dehydrated in a graded series of 20%, 30%, 50% ethanol for 20 min each and maintained in 70% ethanol before measurement. Number of micropyles located on the egg shell was determined by photographing the top area with scanning electron microscope (SEM).

For SEM photographing, the sample preparations were following the methods described by Hao *et al*.^49^. Samples were dehydrated in a graded series of 80%, 85%, 90%, 95% ethanol for 20 min each and 99.9% ethanol for 30 min twice before being transferred to a mixed solution of ethanol and tert-butanol (3:1, 1:1, and 1:3, by volume) for 15 min each, and finally in 100% tert-butanol for 30 min. After removal from the tert-butanol, the specimens were transferred into a freeze-drier (VFD-21S, SHINKKU VD, Japan) for 3 h. The dried specimens were mounted on aluminum stubs using double-sided copper sticky tape and coated with gold/palladium (40/60) in a high-resolution sputter coater (MSP-1S, SHINKKU VD, Japan). The samples were subsequently photographed with a Hitachi S-3400N SEM (Hitachi, Tokyo, Japan) operated at 15 kV. After then, number of micropyle located on the top area of each egg was counted from the images.

### Data analysis

Before analysis, all data were performed with Bartlett’s test and Shapiro-Wilk normality test. For a given parameter, one-way analysis of variance (ANOVA) was applied for comparing the differences among different treatments, while the non-parametric Kruskal-Wallis test was used under non-normal and heteroscedastic conditions. Specially, in delayed mating experiments, at first, the number of micropyles located on eggs collected at the 2^nd^, 5^th^, and 10^th^ day post the first oviposition were compared, and no significant variance was detected among the three sampling periods, with one exception of DDM-C (Control: Kruskal-Wallis χ^2^ = 3.282, *p* = 0.1938; Virgin female: *F*_2, 89_ = 1.323, *p* = 0.272; DDM-A: Kruskal-Wallis χ^2^ = 1.487, *p* = 0.475; DDM-B: *F*_2, 89_ = 1.882, *p* = 0.158; DDM-C: Kruskal-Wallis χ^2^ = 6.574, *p* = 0.0374; DDM-D: *F*_2, 89_ = 0.430, *p* = 0.652), thus these data were pooled together for analysis; in crossing experiments of HAM and HAW, the differences of number of micropyle among the four groups and virgin HAW female (eggs collected at the 5^th^ day post first oviposition) were compared. Means were separated with Tukey HSD test (*p* < 0.05). For analyzing the differences of micropyles, almost 90 available photographs were used in each treatment. All data analyses were conducted with R version 3.2.1 software^50^.

## Data Availability

Te datasets generated and analyzed during the current study are available in the fgshare repository, https://figshare.com/s/ae454eb19c3092e68992.
